# 1106. Case Study. Navigating Burn Surgery and ECMO: A Multidisciplinary Approach to Refractory Respiratory Failure

**DOI:** 10.1093/jbcr/irag033.210

**Published:** 2026-04-06

**Authors:** Cindy Cruz Alvarez, Gabriela Aguiluz

**Affiliations:** Jacobi Burn Center, Union City, NJ; Jacobi Medical Center, Bronx, NY

## Abstract

**Patient Presentation (age range, injury details, relevant history):**

Three patients [30–40]-year-old male with no significant past medical history sustained approximately [40–50% TBSA] deep partial- and full-thickness burns to the torso, face, and upper extremities following a structural fire. They all required early intubation for inhalation injury and developed severe ARDS refractory to maximal ventilatory support.

**Clinical Challenges:**

The clinical course was complicated by progressive hypoxemia, need for serial operative debridements and grafting, and the heightened risk of bleeding and infection with extracorporeal membrane oxygenation (ECMO). Balancing anticoagulation to maintain circuit patency while minimizing surgical bleeding risk was a central challenge.

**Management Approach:**

Veno-venous ECMO was initiated on hospital stay as salvage therapy. Anticoagulation was carefully titrated, and a multidisciplinary team coordinated perioperative management to enable safe operative interventions during ECMO support.

**Outcomes:**

Patients tolerated multiple burn procedures while on ECMO, without catastrophic hemorrhage or infectious complications. They were successfully decannulated and discharged to rehabilitation with preserved pulmonary function.

**Lessons Learned:**

The cases highlight the feasibility of ECMO in burn patients when supported by careful anticoagulation strategies and close interdisciplinary collaboration.

**Applicability to Practice:**

Although ECMO remains controversial in the burn population, these cases demonstrates that with tailored management and multidisciplinary care, it can serve as a life-saving therapy for patients with inhalation injury and refractory ARDS. Broader studies are warranted to refine patient selection and best practices.

